# Comprehensive phenotypic analysis of multiple gene deletions of α-glucan synthase and Crh-transglycosylase gene families in *Aspergillus niger* highlighting the versatility of the fungal cell wall

**DOI:** 10.1016/j.tcsw.2025.100141

**Published:** 2025-01-31

**Authors:** Katharina J. Ost, Mark Arentshorst, Bruno M. Moerschbacher, Mareike E. Dirks-Hofmeister, Arthur F.J. Ram

**Affiliations:** a Münster University, Institute for Biology and Biotechnology of Plants, Schlossplatz 8, 48143 Münster, Germany; b Osnabrück University of Applied Sciences, Faculty of Agricultural Sciences and Landscape Architecture, Laboratory for Food Biotechnology, Oldenburger Landstraße 62, 49090 Osnabrück, Germany; c Leiden University, Institute of Biology Leiden, Fungal Genetics and Biotechnology, Sylviusweg 7, 2333, BE, Leiden, the Netherlands

**Keywords:** Ags α-glucan synthases, Crh transglycosylases, Aspergillus niger, Fungal cell wall, Growth morphology

## Abstract

Multiple paralogs are found in the fungal genomes for several genes that encode proteins involved in cell wall biosynthesis. The genome of *A. niger* contains five genes encoding putative α-1,3-glucan synthases (AgsA-E) and seven genes encoding putative glucan-chitin crosslinking enzymes (CrhA-G). Here, we systematically studied the effects of the deletion of single (*agsA* or *agsE*), double (*agsA* and *agsE)*, or all five *ags* genes (*agsA-E*) present in *A. niger*. Morphological and biochemical analysis of *ags* mutants emphasizes the important role of *agsE* in cell wall integrity, while deletion of other *ags* genes had minimal impact. Loss of *agsE* compromised cell wall integrity and altered pellet morphology in liquid cultures.

Previous studies have indicated that deletion of all *crh* genes in *A. niger* did not result in cell wall integrity-related phenotypes. To determine whether the *ags* and *crh* gene families have redundant functions, both gene families were deleted using iterative CRISPR/Cas9 mediated genome editing. The 12-fold deletion mutant was viable and did not exhibit growth defects under non-stressing growth conditions. A synergistic effect on cell wall integrity was observed in this 12-fold deletion mutant, particularly when exposed to cell wall-perturbing compounds. The cell wall composition, extractability of glucans by alkali, and scanning electron microscopy analysis showed no differences between the parental strain and mutants lacking *ags* genes, *crh* genes, or both. These observations underscore the ability of fungal cells to adapt and secure cell wall integrity, even when two entire cell wall protein-encoding gene families are missing.

## 1 Introduction

The fungal cell wall is a complex dynamic structure primarily composed of chitin, β-glucan, and α-glucan, which form a robust matrix (Latgé, 2007; Free, 2013; Yoshimi et al., 2016, Yoshimi et al., 2017; Gow and Munro, 2017). Chitin, composed of *N*-acetylglucosamine units linked by β-1,4 glycosidic bonds and cross-linked with various glucans, constitutes the rigid core of the wall. Beta-glucans, mostly insoluble and strong triple-helix structures, can be linear or branched, often cross-linked through β-1,3 and β-1,6 linkages or mixed with α-bonds (Latgé, 2010, Latgé, 2020; Yoshimi et al., 2016, Yoshimi et al., 2017; Ruiz-Herrera and Ortiz-Castellanos, 2019). Alpha-glucans, mainly featuring α-1,3 and α-1,4 linkages, interact with the environment and influences immune recognition through the outer components including cell wall (gluco)mannan-proteins, galactomannans, and galactosaminogalactans (Beauvais and Latgé, 2018; Kang et al., 2018; Miyazawa et al., 2020; Chakraborty et al., 2021). In *A. fumigatus* and *S. commune,* it has been shown that α-1,3-glucan is also a major part of the inner rigid polypeptide core of the cell wall. This also holds ture for β-glucans (Kang et al., 2018; Ehren et al., 2020; Safeer et al., 2023). Furthermore, it has been shown that the outer surface is highly dynamic and distinct from the inner layer, with α-glucan and β-glucan localized within the outer mobile shell (Kang et al., 2018; Ehren et al., 2020; Safeer et al., 2023). The presence of α-1,3-glucans in these two distinct layers of the cell wall highlights the structural and functional versatility of this biomolecule. Although these components are common, significant variations in polysaccharide composition and cell wall structure exist among different filamentous fungi (Bowman and Free, 2006; Cabib et al., 2007; Kang et al., 2018; Gow and Munro, 2017; Fernando et al., 2021; Ehren et al., 2020). Additionally, the fungal cell wall contains highly glycosylated proteins including structural proteins and enzymes, which are essential for cell wall synthesis and repair (Bernard and Latgé, 2001; Latgé, 2010; Gow and Munro, 2017; Beauvais and Latgé 2018). Biosynthesis, modification, remodeling, and degradation of the cell wall involves various genes that are activated through the cell wall integrity (CWI) signaling pathway and are often induced under specific conditions (Levin, 2005; Damveld et al., 2008; Yoshimi et al., 2016; Kiss et al., 2019; Wagener et al., 2020).

Congo Red hypersensitive enzymes (Crh enzymes, EC 3.2.1.210) are cell wall-related transglycosylases that convey chitin-β-glucan cross-linking and have been extensively studied in *Saccharomyces cerevisiae* and *Aspergillus niger* (Arroyo et al., 2016; Fang et al., 2019; van Leeuwe et al., 2020). Research on *crh*-deficient strains has indicated that these crosslinks are vital for cell division and cell wall stability in *S. cerevisiae* (Cabib et al., 2007; Cabib, 2009; Arroyo et al., 2016). However, studies of *Aspergillus* spp. have contradicted their roles in yeast. Deleting the entire *crh* gene family in *Aspergillus fumigatus* and *A. niger* did not significantly affect the cell wall integrity, growth morphology, or sugar composition (Fang et al., 2019; van Leeuwe et al., 2020). van Leeuwe et al. (2020) found that deleting all seven *crh* genes (*crhA-G*) did not weaken the cell wall, trigger the CWI response, or impact growth and development in *A. niger*. The *crh* gene family appears to be relevant for cell wall integrity only in the absence of galactomannan synthase (*ugmA*) or α-glucan synthases (*agsA* *+* *E* or *agsE*) (van Leeuwe et al., 2020).

Alpha 1,3-glucan synthases (Ags proteins, EC 2.4.1.183) are large, multi-domain membrane proteins involved in α-glucan biosynthesis (Hochstenbach et al., 1998; Grün et al., 2005; Damveld et al., 2005). Genome analysis revealed that some filamentous fungi possess one *ags* gene, others have multiple genes, and some species lack genes for α-1,3-glucan synthase (Hochstenbach et al., 1998; Reese et al., 2007; Beauvais et al., 2005; Damveld et al., 2005; Vos et al., 2007). The reasons for multiple copies of *ags* genes in filamentous fungi, the quantity of α-1,3-glucan in fungal cell walls, and the existence of different α-glucan forms synthesized by Ags proteins are not well understood. The role of α-1,3-glucan in fungal cell walls has also been debated, even within the same genus. Disrupting a single α-1,3-glucan synthase gene can be lethal in *S. pombe* but only affects the growth rate and capsule anchoring in *C. neoformans* (Hochstenbach et al., 1998; Reese and Doering, 2003; Maubon et al., 2006). In *A. fumigatus*, a triple *ags* gene knockout mutant showed a significant growth rate reduction (Bernard and Latgé, 2001).

Deletion of three *ags* genes in *A. fumigatus* resulted in a strain characterized by the absence of α-1,3-glucan and increased levels of chitin and β-1,3-glucan, but did not affect conidial germination or vegetative growth (Henry et al., 2012; Beauvais et al., 2013). Disrupting *agsB* in *A. nidulans* significantly altered cell wall composition and increased chitin and β-glucan levels (Yoshimi et al., 2013). The authors also showed that *agsB* is essential for proper cell wall formation and hyphal aggregation in liquid culture, whereas *agsA* plays only a minimal role (Yoshimi et al., 2013, Yoshimi et al., 2017). In addition, deleting *agsB,* increased the exposure of β-glucan on the cell surface, leading to increased sensitivity to cell wall stress-inducing agents (Yoshimi et al., 2017). Similar findings were observed in *A. oryzae*, where disruption of *agsB* led to reduced α-1,3-glucan and dispersed hyphae, whereas *agsA* played a minor role. Furthermore, both α-1,3-glucan and galactosaminogalactan (GAG) are crucial for hyphal aggregation, as a double mutant lacking both showed complete dispersion in liquid cultures (Miyazawa et al., 2018, Miyazawa et al., 2020). Additionally, *A. niger* expression data indicated that *ags* genes are induced during various developmental phases and are differentially expressed upon exposure to cell wall-perturbing compounds (Damveld et al., 2005; Jørgensen et al., 2020).

The genome of *A. niger* NRRL3 (ATCC 9029, CBS 120.49) contains five conserved genes encoding putative α-1,3-glucan synthases: *agsA-E* (Pel et al., 2007)*.* Only *agsA* and *agsE* are highly expressed in response to cell wall stress, whereas *agsB* and *agsD* are not detectable during vegetative growth (Damveld et al., 2005). The *agsD* gene is induced during asexual conidiophore development, and *ΔagsE* shows increased sensitivity to Congo Red (Damveld et al., 2005, Damveld et al., 2008; van Leeuwe et al., 2020). Recently, it was shown that the deletion of *agsE* in *A. niger* had a direct impact on colony morphology by reducing the diameter of micro-colonies in liquid cultures and altering the secretome composition (Lyu et al., 2023b).

It has also been demonstrated that in the absence of α-glucan synthases, several *Aspergillus* species exhibit functional compensatory effects, such as altering cell wall polysaccharide composition by increasing chitin and β-glucan content to preserve cell wall stability and structure , or overexpression of certain *ags* genes to restore α-1,3-glucan synthesis and cell wall integrity (Henry et al., 2012; Yoshimi et al., 2013, Yoshimi et al., 2017; Miyazawa et al., 2018). Thus, although the role of α-glucan in fungi has not yet been fully resolved, its absence affects both natural survival and industrial fermentation (Fontaine et al., 2010; Meyer et al., 2011; Wösten et al., 2013; Yoshimi et al., 2017; Lyu et al., 2023a; Miyazawa et al., 2016, Miyazawa et al., 2024). In this study we addressed the role of α-glucan by performing a detailed phenotypic analysis of *A. niger* mutants lacking all five *ags* genes as well as in combination with deletion of the entire *crh* gene family consisting of seven genes*.* Surprisingly, our findings revealed that even the 12-fold deletion did not affect the growth of the mutant strain, indicating that *A. niger* possesses compensatory mechanisms that ensure survival.

## 2 Materials and methods

### 2.1 Strains, media, and growth conditions

All *A. niger* strains used in this study are listed in Table 1. Strains were cultivated in liquid minimal medium (MM) or complete medium (CM) at 30 °C and 180 rpm overnight, including shake flasks with baffles to increase homogenous mixing. Minimal medium (MM) contained 1 % (w/v) glucose or sucrose, 2 mM MgSO_4_, 1× ASPA+N, 1× Trace element solution, and 1.2 % agar, while CM medium additionally contained 0.1 % (w/v) casamino acids and 0.5 % (w/v) yeast extract, according to Arentshorst et al., 2012.

**Table 1:** *A. niger* strains used in this study for analysis on *ags* gene deletions.

Name	Genotype	Reference
N402	*cspA1, amdS*-	Bos et al., 1988
MA234.1	*cspA1, ΔkusA::DR-amdS-DR*	Park et al., 2016
TLF93	*cspA1, ΔkusA::DR-amdS-DR, ΔagsA*	van Leeuwe et al., 2020
TLF94	*cspA1, ΔkusA::DR-amdS-DR, Δ*agsE	van Leeuwe et al., 2020
TLF95	*cspA1, ΔkusA::DR-amdS-DR, ΔagsA* *+* *E*	van Leeuwe et al., 2020
TLF39	*cspA1, ΔkusA::DR-amdS-DR, ΔcrhA-G*	van Leeuwe et al., 2020
MA946.3	*cspA1, ΔkusA::DR-amdS-DR, ΔagsA-C*	This study
KO1.1	*cspA1, ΔkusA::DR-amdS-DR, ΔagsA-E*	This study
MA947.1	*cspA1, ΔkusA::DR-amdS-DR, ΔcrhA-G, ΔagsA-C*	This study
KO2.2	*cspA1, ΔkusA::DR-amdS-DR, ΔcrhA-G, ΔagsA-E*	This study

Spore inoculations were performed using freshly harvested spores scraped from the surface of the CM agar plates using 10 mL of 0.9 % (w/v) NaCl and a cotton swab. Spores in the spore solution were counted using a Bio-Rad TC20™ Automated Cell Counter and filtered through sterile Myracloth Calbiochem filters to remove mycelial debris. Cultures were incubated for 3–4 d at 30 °C.

### 2.2 Multiple gene knockouts using CRISPR/Cas9

Deletion of the *ags* gene family was carried out in the parental strain MA234.1 (N402 background), and in the seven-fold *crh* knockout strain TLF39 using the CRISPR/Cas9 technique in two steps. Therefore, 5- and 12-fold knockout mutants were generated by constructing the first triple *ags* (*agsA, B, C*) knockout mutants in both backgrounds. In the second round of transformation, the remaining *agsD* and *agsE* genes were deleted.

The gene disruptions were based on offering a target-specific DNA repair fragment and hygromycin selection using a pFC332 plasmid, as previously described (Nødvig et al., 2015). To delete each *ags* gene, a specific sgRNA was designed and cloned into pFC332. The sgRNA expression cassette harboring the 20 bp target spacer was implemented for each *ags* gene, targeting their open reading frames (*agsA:* An04g09890 / NRRL3_07454, *agsB:* An15g07810 / NRRL3_04200, *agsC:* An12g02450 / NRRL3_09002*, agsD*: An02g03260 / NRRL3_05996, and *agsE:* An09g03070 / NRRL3_00248).

**Supplementary Fig. S1:**
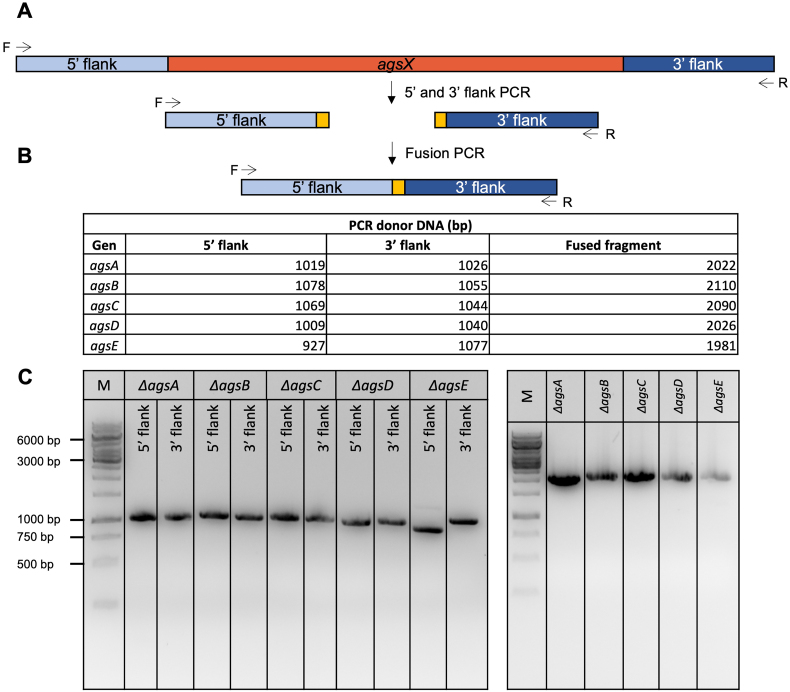
Construction of repair DNA for each *ags* target gene deletion in *A. niger* parental strain (MA234.1) and TLF39 (*Δcrh* genetic background). (A) 5“ and 3‘ flank PCR amplification of region upstream (5’ flank) and downstream (3” flank). Black arrows indicate forward (F) and reverse (R) primer for fragment amplification. (B) Fusion of both flanks with the same primers as indicated above. Table above shows expected fragment size (bp) of single- and fused flanks. (C) Agarose gel (1 %) with successfully amplified 5′ and 3′ flanks (left) and with successfully fused fusion-fragments for *ΔagsA-E* (right) including 1 kb Generuler (M).

In addition, the repair fragments were constructed to remove the entire *ags* ORFs. Generation of sgRNA cassettes and repair fragments was performed using overlap extension PCRs with genomic DNA from wild-type N402 strain. The generated 5‘ and 3’ flanks were fused using target-specific primers containing overlapping sequences. PCR was performed using the templates and primers listed in Supplementary Table S1 and shown in Fig. S1.

**Supplementary Fig. S2:**
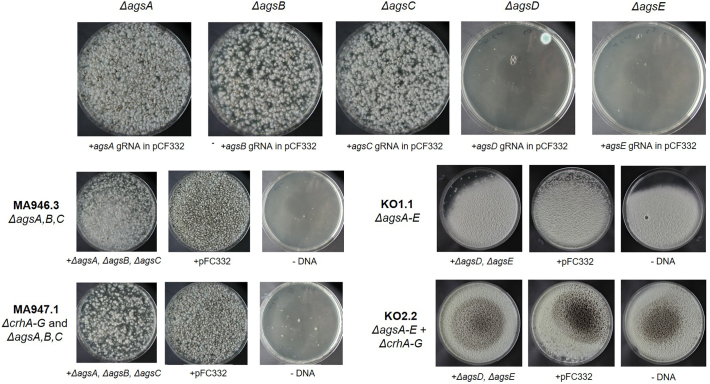
Growth of individual *A. niger* knockout strains MA946.3 (*ΔkusA* background), MA947.1 (*ΔcrhA-G* background), KO1.1 (*ΔagsA-C*) and and KO2.2 (*ΔcrhA-G* + *ΔagsA-C* background) on MMS after PEG-mediated protoplast transformation. Protoplasts were transformed with pFC332 + *agsX* target and repair DNA (left), without repair DNA (center) or without DNA (right). All strains were grown MMS containing 32.5 % sucrose (*w*/*v* %) and 200 μg/mL hygromycin B at 30 °C for 5–7 d.

**Supplementary Fig. S3:**
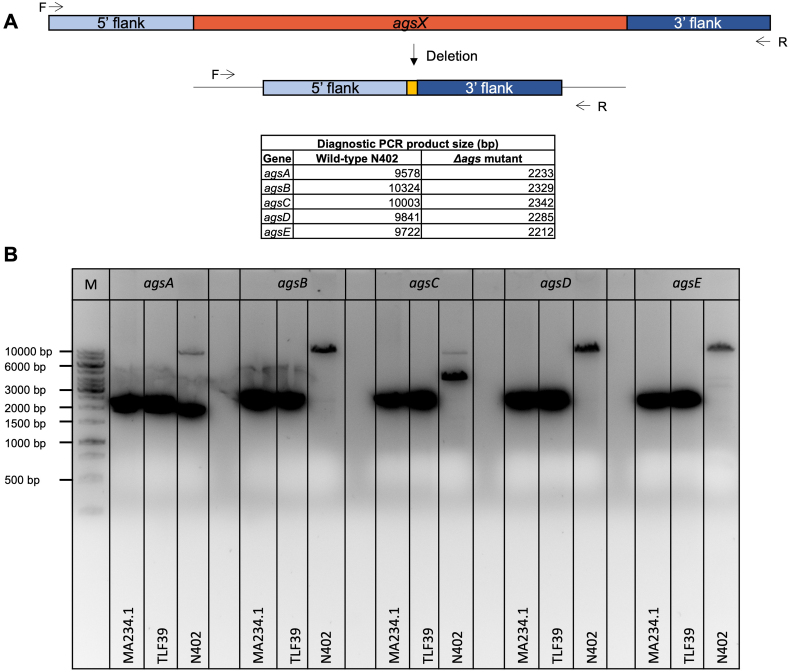
Verification of *ΔagsA-E* gene deletions in *A. niger* MA234.1 and TLF39 (*Δcrh* genetic background) by using diagnostic PCR including N402 as a control. (A) Integration of repair DNA at *agsX* locus for *ags* gene family deletion. Black arrows indicate forward (F) and reverse (R) primer for target amplification. The removed *ags* ORF for each individual *ags* gene in MA234.1 and TLF39 show smaller PCR products compared to N402. The table represents the expected band size (bp) for N402 and both deletion mutants. (B) Agarose gel (1 %) for diagnostic verification including 1 kb Generuler (M).

The vectors were co-transformed with the appropriate DNA repair fragments after protoplastation using the same approach described by Arentshorst et al., 2012. One microgram of DNA was added to the protoplasts for the PEG-mediated transformation. The transformation plates were incubated on MMS for 5–7 d at 30 °C, and the transformed colonies (Fig. S2) were analyzed on selective plates. The first selection step was performed using MMS with 100 μg/mL hygromycin for positive Cas9-sgRNA plasmids. In the second purification step, single colonies were streaked on MMS without hygromycin to allow for the loss of the Cas9-sgRNA plasmid. To double-check if the selection marker was removed, positive transformants were streaked on MM with 100 μg/mL hygromycin. After purification, genotyping was verified using diagnostic PCR (Fig. S3). DNA was extracted as described by Arentshorst et al., 2012.

### 2.3 Expression data analysis

Multi-condition expression data, provided by Prof. Vera Meyer (Department of Applied and Molecular Microbiology, Institute of Biotechnology, Berlin University of Technology, Germany), were analyzed using the method described by van Leeuwe et al., 2020.

Gene enrichment (GO) analysis for each *ags* gene in *A. niger* utilized a gene co-expression network based on transcriptomic datasets from *A. niger* DNA microarray expression studies. Data from the FungiDB functional genomic database (Basenko et al., 2018) were analyzed using available tools from FungiDB, as described by Schäpe et al., 2019, Schäpe et al., 2023.

All data on the DNA microarray platform and gene expression data from 155 different cultivation conditions were extracted from Paege et al., 2016 and Schäpe et al., 2019.

To organize data with enriched GO terms and genes co-expressed in similar biological processes or pathways, the following steps were taken: only over-represented genes with a positive correlation and a Spearman coefficient of 0.5 were analyzed for individual *ags* genes. First, GO terms were evaluated using an adjusted *p*-value cut-off of 0.05. In a subsequent manual sorting step, GO terms with a sample frequency ≥ 60 % (percentage of total samples in which a specific GO term is associated with one or more *ags* genes), result count ≤ 15, and fold enrichment ≥ 50 for each ontology (molecular function, biological process, and cellular component) were selected to identify the most strongly enriched GO terms. Over-represented genes sharing the same GO terms were filtered and analyzed. The manual sorting process removed duplicate GO terms and prioritized the most statistically significant hits in certain pathways based on the higher fold enrichment scores. Unique GO terms for *ags* genes were selected using two criteria: i) GO terms present in 20 % of samples with fold enrichment ≥ 53 to capture highly specific processes and ii) GO terms present in 40–60 % of samples with high background counts and significant *p*-values to uncover potentially overlooked functions.

### 2.4 Cell wall sensitivity assays

The generated strains were exposed to different cell wall-disturbing compounds: Congo Red (CR: 100–800 μL/mL), Calcofluor White (CFW: 100–800 μL/mL), caspofungin (CA, 0.01–0.5 μM), echinocandin B (0.025–1 μM), tunicamycin (1–10 μg/mL), sodium dodecyl sulfate (SDS: 0.004–0.005 %), and hydrogen peroxide (H_2_O_2_:10–50 mM) in MM agar. Spores were spotted with different dilutions of 10^4^, 10^3^, 10^2,^ and 10^1^ spores/mL on MM plates. The plates were incubated for 3–5 d at 30 °C.

### 2.5 Monosaccharide analysis

Monosaccharide analysis, including cell wall isolation, chemical fractionation, dialysis, and subsequent analysis by reversed-phase UHPLC-ESI-MS/MS, was performed according to the methods described by van Leeuwe et al. (2020) with minor modifications.

**Supplementary Fig. S4:**
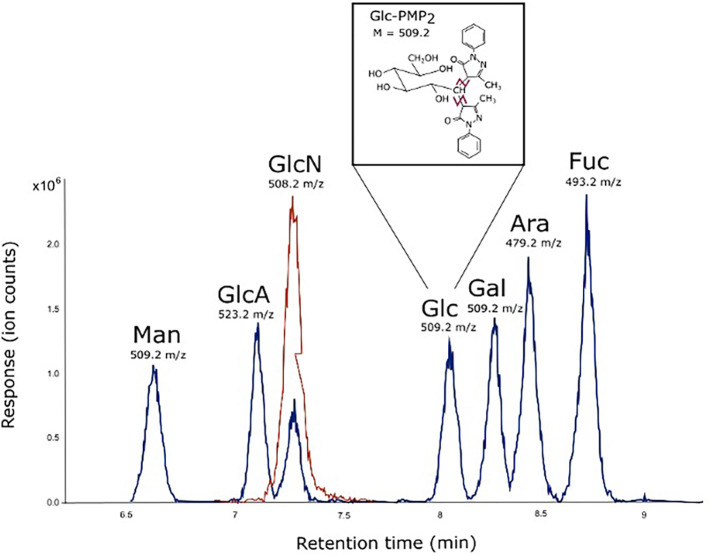
PMP-derivatized monosaccharide standards by using reversed-phase UHPLC-ESI-MS. The extracted ion chromatograms of the PMP_2_-derivatized neural sugars from TFA hydrolysis are shown in blue (mannose Man, glucuronic acid GlcA, glucose Glc, galactose Gal, arabinose Ara, fucose Fuc) and amino sugar from HCl hydrolysis is represented in orange (*N*-glucosamine GlcN) and used for quantification. The fragmentation of given precursor ions is indicated in pink in the structure of Glc-PMP_2_ as an example for MS^2^ spectrum (*m*/*z* = 509.2).

**Supplementary Fig. S5:**
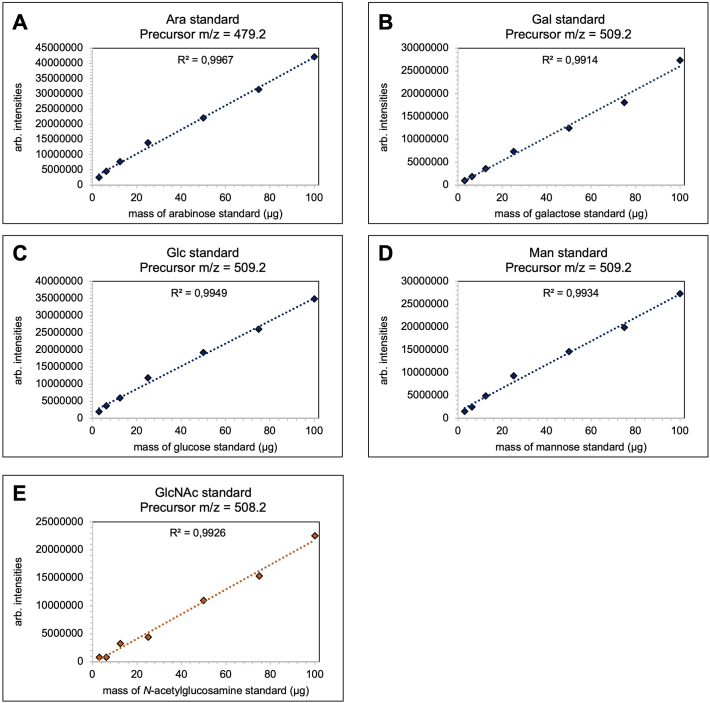
Standard curves for (A) Arabinose (479.2 m/z) by TFA treatment, (B) Galactose (509.2 m/z) by TFA treatment, (C) Glucose (509.2 m/z) by TFA treatment, (D) Mannose (509.2 m/z) by TFA treatment and (E) *N*-acetylglucosamine (508.2 m/z) by HCl treatment. All standards are measured in duplicates for every measurement.

**Supplementary Fig. S6:**
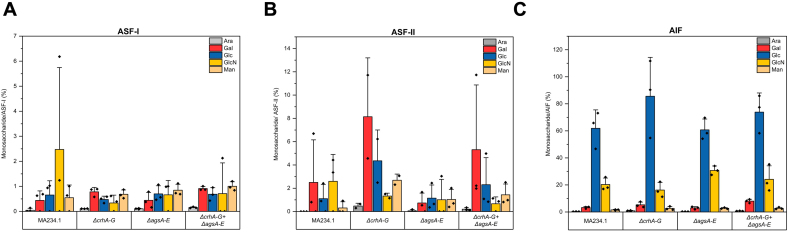
Monosaccharide cell wall composition of parental strain (MA234.1) and multiple gene knockout mutants *ΔcrhA-G*, *ΔagsA-E*, *ΔcrhA-G* + *ΔagsA-E* after chemical fractionation. Detected monosaccharides are arabinose (Ara, gray), galactose (Gal, red), glucose (Glc, blue), *N*-glucosamine (GlcN, yellow), and mannose (Man, orange) according to the Materials and Methods section 2.5. The detected amounts of different monosaccharides are shown in three different fractions, which they were obtained from washed and TFA- or HCl chemical fractionated *A. niger* cell walls. The values for each monosaccharide are shown in percentage shared in each fraction (A) present in alkali insoluble fraction I (ASF-I)*, (B) present in alkali insoluble fraction II (AIF-II)* and (C) present in alkali soluble fraction (AIF). All fractions were measured from three biological replicates, if not indicated differently. Data points are indicated as ◆ and standard deviation is represented by error bars. (*) Fractions are additionally dialyzed after chemical fractionation.

For cell wall isolation, liquid cultures of CM were inoculated with 10^6^ spores/mL and incubated at 30 °C and 200 rpm for 17 h. Harvested mycelia were washed, freeze-dried, and lyophilized. The resulting samples were either chemically fractionated, dialyzed, lyophilized or directly hydrolyzed for analysis (Fig. S6). Monosaccharide standards and cell wall samples were hydrolyzed using either trifluoroacetic acid (TFA) or hydrochloric acid (HCl) and derivatized with phenyl-3-methyl-5-pyrazolone (PMP), as outlined by van Leeuwe et al., 2020. Samples were analyzed using the same reversed-phase UHPLC coupled with an ESI-MS/MS system described by van Leeuwe et al., 2020. The gradient elution profile used for the separation of PMP-derivatized monosaccharides followed the method of Xu et al. (2018), with MS parameters including the Auto MS^2^ mode and CID fragmentation. The peak area of the MS^1^ data was integrated using Bruker Data Analysis Software v4.1, with predefined retention times for each fragment ion intensity for a given precursor ion (Fig. S4). The monosaccharide content in 100 μg of lyophilized cell wall material was recalculated as per van Leeuwe et al. (2020) using calibration curves from PMP-derivatized monosaccharide standards (Fig. S5). HCl hydrolysis converted *N*-acetylglucosamine to glucosamine, which was accounted for in the calculations, and one water molecule per monosaccharide was subtracted to account for the glycosidic linkages in cell wall polymers.

### 2.6 Scanning electron microscopy (SEM)

Biomass from CM liquid cultures of the *A. niger* parental strain and knockout mutants grown at 30 °C and 180 rpm was harvested after 24 h via vacuum filtration, washed three times both with H_2_O and 0.9 % NaCl, and fixed with 2 % glutaraldehyde for 2 h at 4 °C. After fixation, samples were washed twice with 70 % ethanol and stored in 70 % acetone.

Scanning electron microscopy (SEM), including sample mounting, dehydration, and coating, was conducted using the IBL Microscopy Facility at Leiden University. Samples were dehydrated (70 % acetone for 15 min, 80 % acetone for 15 min, 90 % acetone for 15 min, and 100 % acetone for 15 min) and critical point dried with CO_2_ (Baltec CPD-030).

Dried pellets were placed on stubs with carbon tape, coated with platinum‑palladium (80/20) using a gold sputter coater, and imaged using a JEOL JSM7600.

### 2.7 Particle size determination of cultures grown in liquid shake flasks

**Supplementary Fig. S7:**
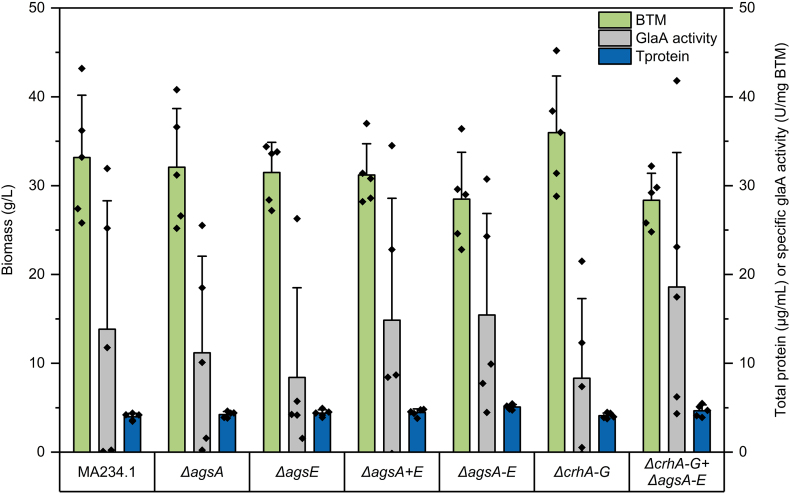
Biomass formation, protein production and specific glucoamylase activity in liquid shake flask cultivation of parental strain (MA234.1) and knockout mutants *ΔagsA*, *ΔagsE*, *ΔagsA + E*, *ΔagsA-E*, *ΔcrhA-G*, *ΔcrhA-G* + *ΔagsA-E*. Cultivation was performed in MM with 10 g/L glucose and 100 g/L maltodextrin after 8 d at 30 °C and 180 rpm. Cultures were inoculated with freshly prepared spores and a final concentration of 10^6^ spores/mL. Green colour shows formed biomass (g/L), grew colour demonstrates the quantified specific glucoamylase (glaA) activity (U/mg BTM), and blue colour represents the total amount of proteins released in the supernatant (μg/mL) in five independent experiments. Each biological replicate is shown as individual data point (◆) and standard deviation is represented by error bars.

All strains were cultured in shake flasks with 20 % CM, MMG, or MMG containing 100 g/L maltodextrin at 30 °C and 180 rpm. Cultures were inoculated with 10^6^ spores/mL, and images were taken after 2, 4, 6, and 8 d. The samples were further analyzed for biomass formation, protein production, and glucoamylase activity in the mycelia-free supernatant (Fig. S7).

The particle size of each microcolony from the parental strain and various *ags* mutants was quantified using ImageJ Volume 1.53 t (Schneider et al., 2012). After 18 h of cultivation in complete medium at 30 °C and 180 rpm, the biomass was washed thrice with H_2_O and once with 0.9 % NaCl. The washed microcolonies were transferred to a Petri dish and submerged in water for top-view photography. The particles were defined according to their diameters. The ImageJ calibration matched pixels to physical dimensions using a petri dish diameter (8 cm) as a reference. Images were converted to a binary format by thresholding, setting size limits to analyze mycelial particles in a segmented image.

Additional settings ensuring complete particle structures were included, whereas voids within particles and post-thresholding were excluded. Segmented image analysis provided data on the particle count, distribution, area, and average microcolony size. Analysis was performed using a total of ≥85 pellets for each strain. Particle area was analyzed using one-way ANOVA followed by Tukey post-hoc test for multiple comparisons (*p* ≤ 0.05).

### 2.8 Colony morphology on agar plates

Spores were harvested from CM agar plates incubated for 3 d at 30 °C and automatically counted as described above. For colony morphology analysis on agar plates, 5 μL of 2 × 10^7^ spores/mL of each strain was dropped in the center of the CM agar plates. The morphology, colony diameter, and growth rate over 4 d were measured in triplicate at 30 °C.

Fungal growth rate (μ) was assessed by measuring the expansion of colonies over time. The specific growth rate was expressed in d^−1^ (per day) and calculated using the following equation:

(1) $$\mu =\ln\left(x_{1}\right)-\ln\left(x_{2}\right)/t_{2}-t_{1}$$

where x_1_ and x_2_ represent colony diameters on days 1 (t_1_) and 4 (t_2_), respectively. The area of the colonies was calculated using the formulae below,

(2) $$A={\left(d/2\right)}^{2}\times \pi$$

assuming that the colonies were circular. Colony area for each day was analyzed using one-way ANOVA followed by Tukey post-hoc test for multiple comparisons (*p* ≤ 0.05) and is represented in Supplementary Table S3.

## 3 Results

### 3.1 Iterative CRISPR/Cas9-mediated genome editing for functional studies of the *ags* gene family in *A. niger*

For the construction of *A. niger* strains with single, double, and five-fold *ags* gene deletions, a marker-free CRISPR/Cas9-based genome editing method was used (Nødvig et al., 2015; Song et al., 2018; van Leeuwe et al., 2019). The *ags* gene deletions were carried out in an iterative manner (single, double, and triple gene deletions in one transformation). A DNA repair fragment was provided during transformation to be integrated into the genome of *A. niger* via homologous recombination (HR) to repair the Cas9-induced double strains break. Starting with *A. niger* parental strain MA234.1 (*Δku70* in the N402 background) or 7-fold *crh* deletion mutant (TLF39 from van Leeuwe et al., 2019, van Leeuwe et al., 2020), the strains were constructed as shown in Table 1. Strain KO1.1 and KO2.2, containing the five *ags* genes deleted (*ΔagsA-E*) or the five *ags* genes in combination with the seven *crh* genes deleted (*ΔcrhA-G* *+* *ΔagsA-E*), were used for further studies.

### 3.2 Expression data show that *agsA*, *agsC*, and *agsE* are the most strongly induced genes at different growth conditions

*Ags* genes exhibit varying expression levels in response to cell wall stress and during different fungal growth phases (Park et al., 2016; Damveld et al., 2005; Jørgensen et al., 2020).

Notably, *agsE* was identified as the most highly expressed α-1,3-glucan synthase, whereas *agsA* showed the strongest reaction to cell wall stress (Damveld et al., 2005). To further extend this analysis, we examined the expression of all five *ags* genes across multiple growth conditions and developmental stages using a microarray dataset (Paege et al., 2016; Schäpe et al., 2019, Schäpe et al., 2023). Highlights of varying conditions and growth phases, including different carbon sources, antifungal exposure, conidial germination, and sclerotium formation, are presented in Table 2.

**Table 2:**
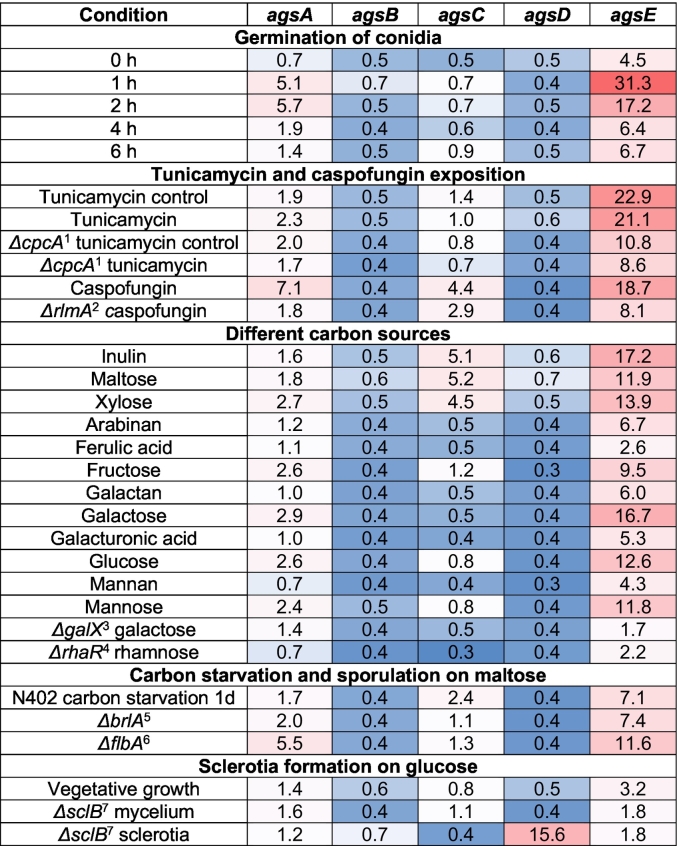
Microarray expression data of *ags* genes as relative to actin under the respective conditions. Colour-coding represents gene expression levels: blue (low; minimum set at 0.3 %), white to rose (medium), and red (high; maximum set at 31.3 %). The analysis was performed on data published by Paege et al., 2016 and Schäpe et al., 2019.

^1^*cpcA*: Basic leucine zipper transcription factor of cross-pathway control (An01g07900); ^2^*rlmA*: Conserved MADS box transcription factor of CWI pathway (An02g12210); ^3^*galX*: Putative zinc-binding transcription factor of galactose catabolism via oxidoreductive pathway (An16g01640); ^4^*rhaR*: Putative transcription factor of rhamnose utilization (An13g00930); ^5^*brlA*: Putative transcription factor of conidiophore development (An01g10540); ^6^*flbA*: Putative regulator of G-protein signaling (An02g03160); ^7^*sclB*: Transcription factor of sclerotial formation and asexual reproduction (An01g06620).

*AgsA* and *agsE* were highly expressed during the first two hours of germination and vegetative growth when exposed to caspofungin, as well as under non-perturbed growth conditions with fructose, galactose, glucose, and mannose. Additionally, carbon sources, such as inulin, maltose, and xylose, enhanced *agsA*, *agsC*, and *agsE* expression after 8 h (data extracted from Yuan et al., 2006; Jørgensen et al., 2009). The analysis showed a stronger response of *agsA*, *agsC*, and *agsE* to the cell wall-stressing compound caspofungin than to tunicamycin or its mutants (Meyer et al., 2007). Notably, the non-sporulating *Δflb*A mutant exhibited a different morphology and strong expression of *agsA* and *agsE* during carbon starvation on maltose after 1 d (data extracted from Nitsche et al., 2012; Krijgsheld et al., 2013). Furthermore, *agsD* was highly and specifically expressed during sclerotia formation (data extracted from Jørgensen et al., 2020).

Our extensive analysis of multiple gene expression datasets revealed that *agsA*, *agsC*, and *agsE* were expressed under similar conditions, with *agsE* being the most highly expressed gene under the tested cultivation conditions. The data also reiterate that *agsD* is only active during sclerotium development, whereas the function of *agsB* remains unclear.

### 3.3 Co-expression analysis showed that the *ags* genes form different co-expression networks

**Fig. 1:**
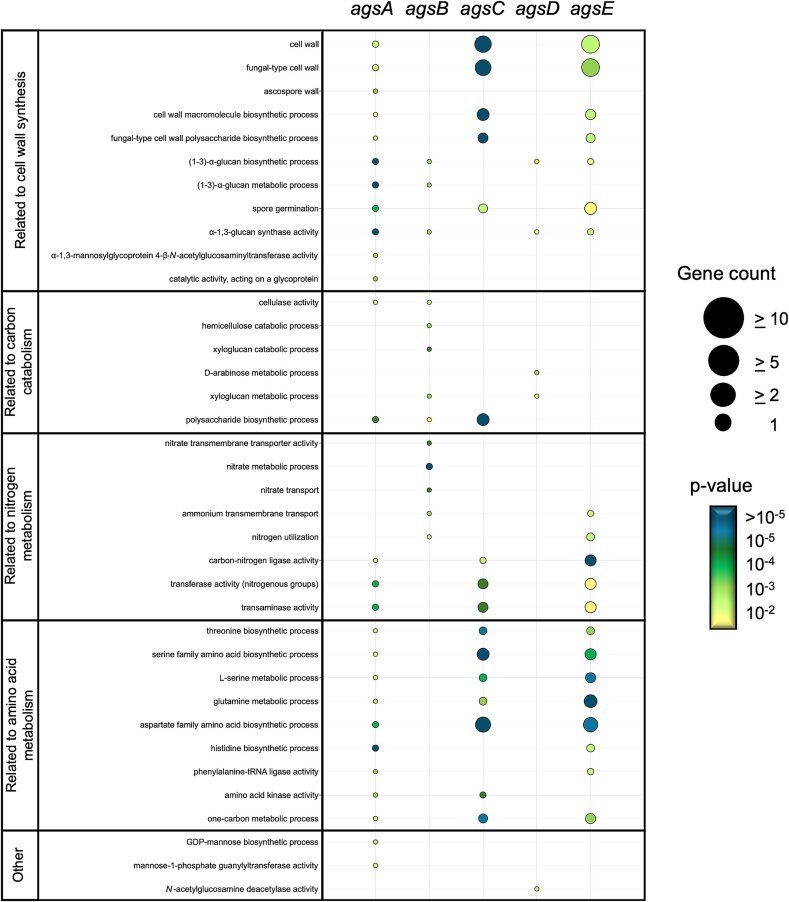
Gene ontology (GO) enrichment analysis in *A. niger* based on transcriptomic datasets and gene co-expression network data from Schäpe et al., 2019, Schäpe et al., 2023. Co-expressed genes for the individual *ags* genes were identified only if a positive correlation was found and a Spearman coefficient of at least 0.5. The results were further narrowed down to a *p*-value cutoff of 0.05. Resulting from this, only unique and over-represented *ags* genes for each ontology of certain biological processes and pathways for selected GO terms are shown.

To identify specific gene networks and further understand the role of individual Ags proteins, a gene co-expression analysis followed by Gene Ontology (GO) enrichment analysis (Table S2) was conducted using tools developed by Schäpe and colleagues (Schäpe et al., 2019, Schäpe et al., 2023). These tools are available in FungiDB (Basenko et al., 2018). The co-expression analysis for individual *ags* genes, using threshold values defined in Materials and Methods section 2.3, revealed 24 (*agsA*), 19 (*agsB*), 310 (*agsC*), 133 (*agsD*), and 979 (*agsE*) co-expressed genes. GO term enrichment analysis of the co-expressed genes identified the most significantly enriched GO terms, detailing the number of genes associated with each *ags* gene in each ontology. The focus was on identifying over-represented GO terms exclusively linked to a single *ags* gene, thus providing insights into their potential roles in biological processes or pathways. A selection of GO terms linked to either multiple or single *ags* gene(s) and prioritized by statistical significance is shown in Fig. 1.

The data confirmed that *agsA* and *agsE* were over-represented in GO terms related to cell wall biosynthesis, particularly α-1,3-glucan production (GO:0047657; GO:0070596), with *ags* genes *A*, *C*, and *E* sharing similar terms, such as polysaccharide/macromolecule biosynthesis (GO:0000271; GO:0070596; GO:0051278; GO:0044038), fungal-type cell wall (GO:0005618; GO:0009277), and amino acid biosynthesis processes (GO:0009067; GO:0000105; GO:0009070; GO:0009088). The *agsA* gene was uniquely associated with mannose-biosynthetic/−metabolic processes (GO:0019673; GO:0009298) and cellulase activity (GO:0008810), whereas *agsB* was over-represented in nitrogen utilization (GO:0019740), nitrate assimilation (GO:0042128), nitrate metabolic process (GO:0042126), nitrate transport (GO:0015112; GO:0015706; GO:0016769), and co-expressed with genes involved in hemicellulose and xyloglucan catabolic processes (GO:2000895; GO:2000899). *AgsD* was enriched in arabinose/arabitol catabolic and metabolic processes (GO:0019571; GO:0046372) and *N*-acetylglucosamine deacetylase activity (GO:0050119).

The analysis revealed that only a few GO terms and genes were significantly over-represented with *ags* genes, primarily those associated with the production and transformation of biological molecules within cellular function, growth, and maintenance.

Notably, no genes directly related to α-1,3-glucan biosynthesis were identified for *agsC*, and significant GO terms related to nitrogen metabolism were found only for *agsB*.

The GO enrichment analysis indicated a correlation between the carbon and nitrogen processes and the expression of certain *ags* genes. The expression data clearly demonstrated that the different *ags* genes have distinct expression patterns.

This suggests that the presence of five Ags-encoding genes likely allows the cell to regulate and control molecules involved in cellular function, growth, and maintenance. Notably, no genes were found to be directly related to α-glucan synthesis under specific conditions.

### 3.4 Increased sensitivity towards cell wall perturbing compounds after *agsE* gene deletion

**Fig. 2:**
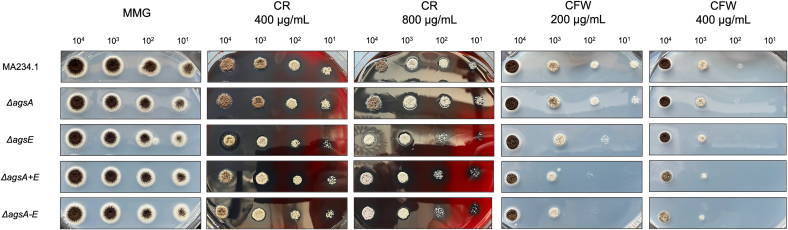
Sensitivity assay on Congo Red (CR) and CalcoFluor White (CFW) at two different concentrations, respectively. Parental strain (MA234.1) and knockout mutants *ΔagsA*, *ΔagsE*, *ΔagsA* *+* *E*, and *ΔagsA-E* were grown on MM-400/−800 μg/mL CR and MM-200/−400 μg/mL CFW. All strains were inoculated with the same concentration of spores from left to right 10^4^, 10^3^, 10^2^, 10^1^ spores/mL. As a reference, all strains were additionally grown on MMG. The plates were incubated at 30 °C for 72 h. (For interpretation of the references to colour in this figure legend, the reader is referred to the web version of this article.)

Given the anticipated role of Ags proteins in maintaining cell wall integrity through α-glucan synthesis, *ags* knockout mutants are expected to show increased sensitivity to cell wall-disrupting compounds, such as Congo Red (CR) and CalcoFluor White (CFW). Thus, we have examined growth phenotypes of the *ags* deletion strains compared to the parental *A. niger* strain (MA234.1). The growth of the parental strain and *ags* mutants was analyzed under non-stressed conditions (MM, with glucose (MMG) and the strains were tested for hypersensitivity towards CR (400 μg/mL and 800 μg/mL) and CFW (200 μg/mL and 400 μg/mL) (Fig. 2).

The mutant strains exhibited no growth or sporulation defects on MMG when compared to the parental strain. Both the parental strain and the mutants showed altered colony morphology with a visible radial growth reduction and non-sporulation effects that increased at higher concentrations of the drugs used, but no hypersensitive or resistance was observed for any of the mutants when compared to the parental strain (Fig. 2).

Notably, no genes were directly related to *ags-*knockout mutants under varying CR and CFW concentrations. Deleting the *agsE* gene increased sensitivity to Congo Red and CalcoFluor White, whereas *ΔagsA* showed sensitivity similar to that of the parental strain, suggesting that *agsA* has a limited role in antifungal sensitivity. A slight difference in colony size was observed on CR-plates for the *ΔagsE, ΔagsA + E* and *ΔagsA-E* mutant strains compared to the parental strain and the *ΔagsA* mutant at 10^1^ spores/mL inoculations*.* Interestingly, higher sensitivity to CFW was observed when *ΔagsE* was combined with *agsA* deletion (*ΔagsA + E*) or when the entire *ags* gene family was removed (*ΔagsA-E*).

In summary, removing *agsE* increases sensitivity to CR and CFW, whereas *ΔagsA* had no notable impact on growth, sensitivity to the cell wall disturbing compound CFW and CR or sporulation.

Deleting the entire *ags* gene family did not further affect cell wall integrity compared to the *agsE* single or double knockout mutants (*ΔagsE* or *ΔagsA +*), suggesting the unique roles of individual *ags* genes under cell wall stress conditions.

### 3.5 Synergistic effect after the loss of *ags* and *crh* gene families

Van Leeuwe et al. 2020 showed that the deletion of all seven members of chitin-β-1,3-glucan cross-linking (Crh) enzymes in *A. niger* did not lead to growth limitations on cell wall interrupting compounds whereas the deletion of *agsE* had a drastic effect on colony size and sporulation on CR. To determine whether the *ags* and *crh* gene families possess redundant functions, a single strain was generated in which both gene families were deleted (*agsA-E* and *crhA-G*).

**Fig. 3:**
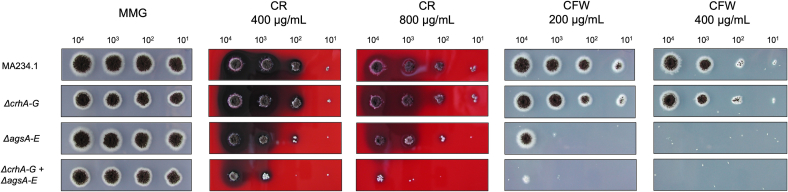
Sensitivity assay on Congo Red (CR) and CalcoFluor White (CFW) at two different concentrations, respectively. Parental strain (MA234.1) and knockout mutants *ΔcrhA-G*, *ΔagsA-E*, and *ΔcrhA-G* + *ΔagsA-E* were grown on MM-400/−800 μg/mL CR and MM-200/−400 μg/mL CFW. All strains were inoculated with the same concentration of spores from left to right 10^4^, 10^3^, 10^2^, 10^1^ spores/mL. As a reference, all strains were additionally grown on MMG. The plates were incubated at 30 °C for 96 h. (For interpretation of the references to colour in this figure legend, the reader is referred to the web version of this article.)

**Fig. 4:**
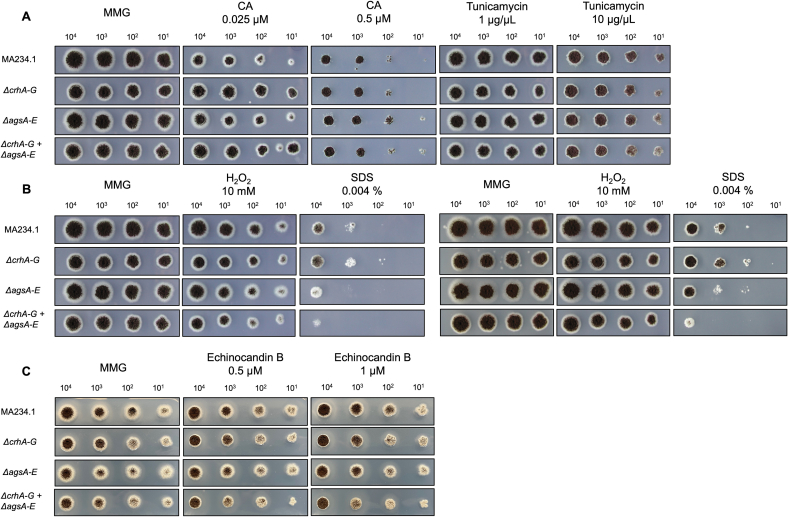
Sensitivity assay on different cell wall stressing compounds at 30 °C. Parental strain (MA234.1) and knockout mutants *ΔcrhA-G*, *ΔagsA-E*, *ΔcrhA-G* + *ΔagsA-E* were grown on: (A) MMG-0.025/0.5 μM caspofungin, MMG-1/10 μg/μL tunicamycin after 96 h. (B) MMG-10 mM H_2_O_2_ and MMG-0.004 % SDS for 96 h (left) and 120 h (right). (C) MMG-0.5/1 μM echinocandin B for 72 h. All strains were inoculated with the same concentration of spores from left to right 10^4^, 10^3^, 10^2^, 10^1^ spores/mL. As a reference, all strains were additionally grown on MMG.

This strain still grew normal under non-stress conditions, so that we conclude that vegetative growth and overall condition of the strain are surprisingly not severely affected based on our assay (Fig. 3). However, when investigating the impact of exposure to the cell wall-perturbing compounds CR and CFW, the 12-fold knockout strain lacking both the *ags* and *crh* gene families was almost unable to grow (Fig. 3). This suggests that the combined absence of both gene families has a more pronounced impact compared to *ΔagsA-E* knockout and *ΔcrhA-G*. Extending the sensitivity assays to further cell wall- and cell membrane-disrupting agents, such as sodium dodecyl sulfate (SDS, strong detergent), hydrogen peroxide (H_2_O_2_, induces oxidative stress), tunicamycin (inhibits *N*-glycosylation, induces unfolded protein response), caspofungin (CA), and echinocandin B (both inhibit β-glucan biosynthesis),

allowed us to further analyze the effects and differences of the entire *ags*-gene family deletion, both individually and in combination with *Δcrh* (Fig. 4). These experiments revealed that both *ΔagsA-E* strain and *ΔcrhA-G* strain did not affect growth on CA, tunicamycin, echinocandin B, and hydrogen peroxide, but showed only a clearly more sensitive phenotype only when grown on 0.004 % SDS.

Taken together, the increased sensitivity to cell wall-disrupting compounds such as Congo Red, CalcoFluor White, and SDS was higher when both gene family deletions were combined. However, sensitivity tests against known fungal cell wall synthesis inhibitors, such as caspofungin, tunicamycin, and echinocandin, demonstrate no increased sensitivity towards *A. niger* mutants lacking both α-1,3-glucan biosynthetic and chitin-β-glucan cross-linking enzymes*.*

### 3.6 No alteration in sugar composition between parental and *ags* mutants

Reorganization of cell wall composition in response to genetic modifications or environmental factors is a known adaptation process in pathogenic fungi and plants (Zhao et al., 2020; Chakraborty et al., 2021).

**Fig. 5:**
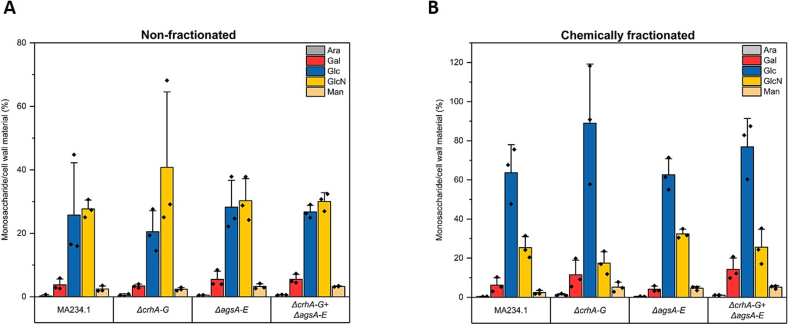
Monosaccharide cell wall composition of parental strain (MA234.1) and multiple gene knockout mutants *ΔcrhA-G*, *ΔagsA-E*, *ΔcrhA-G* + *ΔagsA-E*. The detected monosaccharides were arabinose (Ara, gray), galactose (Gal, red), glucose (Glc, blue), N-glucosamine (GlcN, yellow), and mannose (Man, orange), according to the Materials and Methods section 2.5. The detected amounts of different monosaccharides are shown as percentages from (A) non-fractionated and (B) washed and chemically fractionated cell wall material (sum of all obtained fractions: ASF—I, ASF-II, and AIF). Each sample was analyzed using three biological replicates. Data points are indicated as ◆ and standard deviation is represented by error bars. (For interpretation of the references to colour in this figure legend, the reader is referred to the web version of this article.)

For example, gene deletions reshuffled intramolecular dynamics by increasing the amount of β-1,3-glucan and changing water accessibility in *A. fumigatus* (Chakraborty et al., 2021). Therefore, we analyzed the monosaccharide composition of the cell walls of the *ags*- and *crh*-mutant strains (Fig. 5 and S6). To improve the extractability of glucans from cell walls, the samples underwent two distinct alkali treatments according to Materials and Methods section 2.5 before they were derivatized for the detection of neutral and amino sugars through UHPLC-ESI-MS/MS. The results demonstrated no discernible variations in glucose and *N*-acetylglucosamine content between the mutants and the parental strain in the total cell wall fractions (Fig. 5). Further, chemically fractionation of the cell wall resulted in only slight differences in the contents of galactose and glucose in some sub-fractions (Supplementary Fig. S6).

**Fig. 6:**
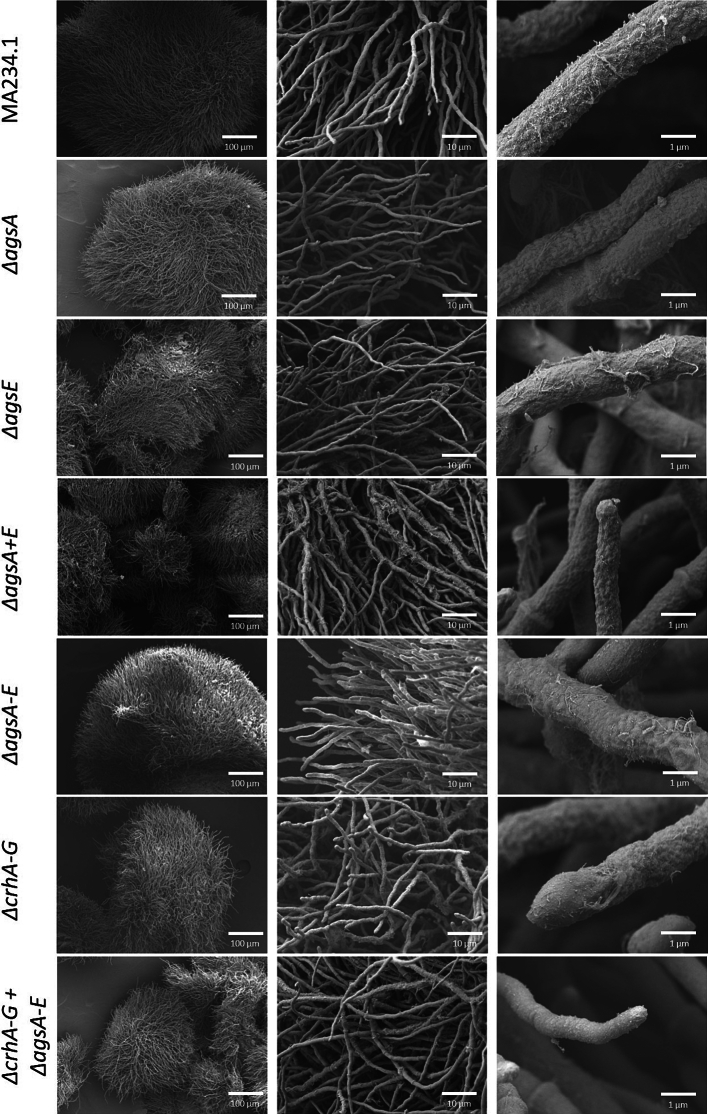
Scanning electron microscopy (SEM) images of the parental strain (MA234.1) and knockout mutants *ΔagsA*, *ΔagsE*, *ΔagsA+E*, *ΔagsA-E*, *ΔcrhA-G*, and *ΔcrhA-G* + *ΔagsA-E*. All strains were grown in CM medium at 30 °C and 180 rpm for 1 d inoculated with 10^6^ spores/mL. Harvested and washed mycelia were fixed with 2 % glutaraldehyde for 2 h. The left column represents the view of the spore (100× magnification), the middle column shows hyphae growth (1000× magnification), and the right column zooms-in to the tips (10′000× magnification).

A closer and more detailed examination of the *ags* mutant cell wall surfaces at the micro-scale was performed using Scanning Electron Microscopy (SEM) (Fig. 6). The SEM analysis revealed no evidence of structural differences in cell wall morphology, hyphal damage, or indications of lysis in either the spores or hyphae of the *ags*-and/or *crh*-deficient mutants when compared to the parental strain.

Taken together, the analysis of monosaccharide composition, glucan extractability, and scanning electron microscopy of the cell wall revealed no differences or reorganization of the major structural polysaccharides after removing *ags* genes, *crh* genes, or both, compared with the parental strain.

### 3.7 Deletion of *agsE* leads to a unique growth phenotype in liquid culture and combining *Δcrh* alters colony morphology

For α-1,3-glucan synthase-deficient strains of *A. niger* and *A. nidulans*, morphological changes characterized by reduced microcolony size a expression data clearly nd aggregation of germinating spores by clumping have been described (Yoshimi et al., 2013; He et al., 2014; Lyu et al., 2023a).

**Fig. 7:**
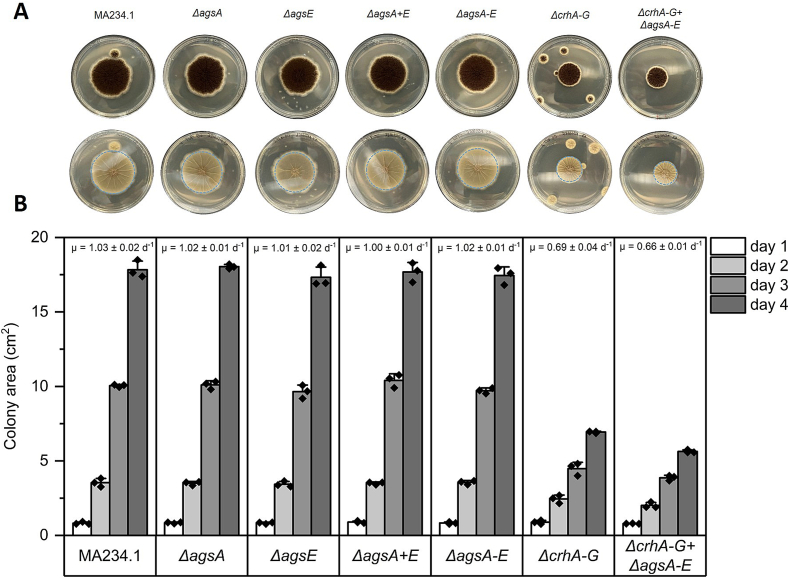
Colony morphology of parental strain (MA234.1) and knockout mutants *ΔagsA*, *ΔagsE*, *ΔagsA+E*, *ΔagsA-E*, *ΔcrhA-G*, and *ΔcrhA-G* + *ΔagsA-E* on CM solid medium. Spores with an inoculum concentration of 2 × 10^5^ spores/mL were grown on CM agar plates for 4 d at 30 °C. As the spores were dropped in the center of the agar plates, the colony area was manually measured every day using the diameter of the colonies. The specific growth rate μ was calculated according to formulae (1) in the Materials and Methods section 2.8. (A) Top (first row) and bottom (second row) views of agar plates after 4 d. The dashed blue circle represents the area used to quantify the diameter. (B) Quantification of colony area (cm^2^) over 4 d of three biological replicates using formulae (2). Data points are indicated as ◆ and standard deviation is represented by error bars. Data were analyzed using one-way ANOVA followed by Tukey's post-hoc test. Statistical significance (*p* ≤ 0.001) was observed only in the strains with *crh* gene deletions (*ΔcrhA-G* and *ΔcrhA-G* + *ΔagsA-E* respectively) at 2,3, and 4 d when compared to the parental strain and other *ags*-deficient strains (see Supplementary Table S3). (For interpretation of the references to colour in this figure legend, the reader is referred to the web version of this article.)

**Fig. 8:**
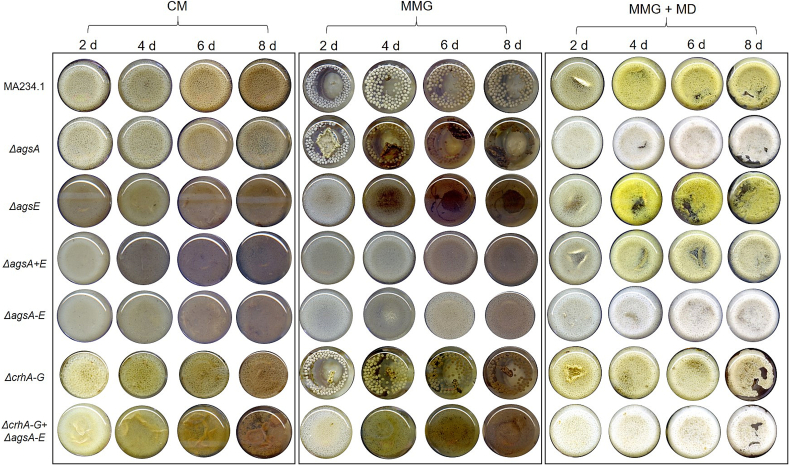
Growth morphology of parental strain (MA234.1) and knockout mutants *ΔagsA*, *ΔagsE*, *ΔagsA* *+* *E*, *ΔagsA-E*, *ΔcrhA-G*, *ΔcrhA-G* + *ΔagsA-E* in different liquid growth medium: (A) Complete medium (CM), (B) minimal medium with 10 g/L glucose (MMG) and (C) MMG containing 100 g/L maltodextrin (MMG + MD). All strains were first grown in 50 mL CM at 30 °C and 180 rpm for 8 d and then transferred to the shown liquid shaken cultures with an inoculated concentration of 10^6^ spores/mL. Images represent the bottom of each shake flask on a given day after incubation.

To determine whether specific *ags* genes are responsible for an individual growth phenotype, or whether growth morphology requires the removal of the entire gene family, we grew the strains under various cultivation conditions, including different cultivation media. Therefore, a detailed examination of colony morphology on solid agar (Fig. 7) and growth morphology in liquid medium (Fig. 8) during vegetative growth of *A. niger* mutants *ΔagsA*, *ΔagsE*, *ΔagsA + E*, *ΔagsA-E*, and *ΔcrhA-G* *+* *ΔagsA-E* was performed. The colony morphology on MMG after deletion of a single *ags* gene (*agsA* or *agsE*) was not altered compared to that of the parental strain (Fig. 7A).

Inactivating all five *ags* genes, however, resulted in fewer sporulating colonies with aberrant growth behavior, particularly at the edges of the colonies. In particular, when *ΔagsA-E* was combined with *ΔcrhA-G*, reduced radial growth and more compact colonies were observed compared to both the parental strain and the mutant lacking *ags* genes. The radial extension rates were determined after growing all strains on CM agar plates and measuring the area of the colonies over four days in biological triplicates (Fig. 7B). The colony characteristics of the mutant lacking both gene families were reproducible on CM agar.

All *ags* gene knockout strains showed comparable radial extension rates compared to the parental strain on CM agar plates. The strains lacking *crh* genes (*ΔcrhA-G)* and the 12-fold mutant (*ΔcrhA-G* *+* *ΔagsA-E)* showed a 33 % and 36 % slower radial extension rate, respectively (μ = 0.69 cm^2^d^−1^ and 0.66 cm^2^d^−1^ compared to 1.03 cm^2^d^−1^ for the parental strain).

**Fig. 9:**
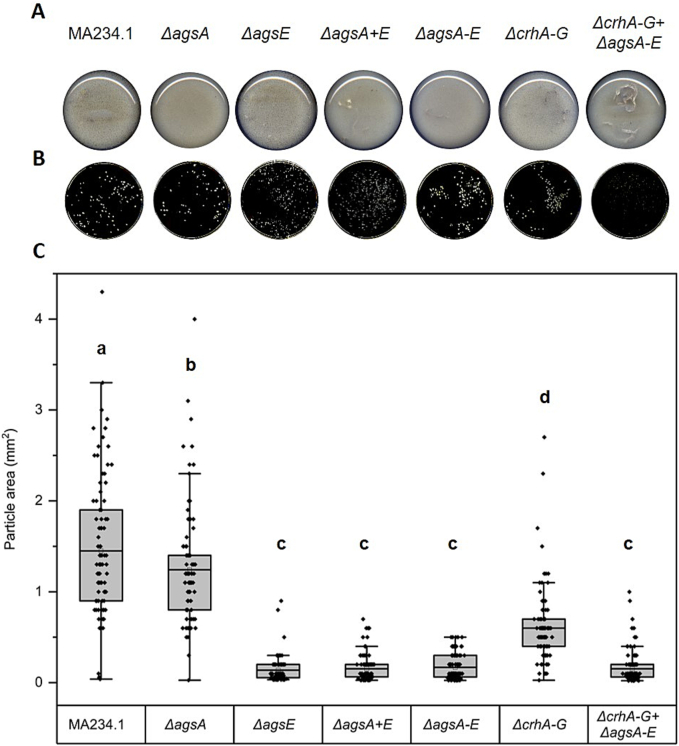
Liquid shake flask cultivation in CM and analysis of particle size of parental strain (MA234.1) and knockout mutants *ΔagsA*, *ΔagsE*, *ΔagsA+E*, *ΔagsA-E*, *ΔcrhA-G*, and *ΔcrhA-G* + *ΔagsA-E* using the ImageJ software. The cultures were inoculated with a final concentration of 10^6^ spores/mL. (A) Images of the bottom of the shake flask cultivation in CM medium at 30 °C and 180 rpm for 1 d. (B) Top view of the washed pellets from these cultures in petri dishes used for particle size determination. (C) Box plot of the calculated particle area (mm^2^) of the washed pellets using the ImageJ software according to the Materials and Methods section 2.7. The average particle area for each strain is shown as a black line in the boxes, each analyzed particle is represented as an individual point (◆) and error bars indicate the particle size distribution. Samples that do not share the same letters indicate statistical significance (p ≤ 0.001), as determined by one-way ANOVA combined with Tukey's post hoc test.

In addition, distinct growth phenotypes were consistently reproducible across various liquid grown cultures (Fig. 8). The parental strain MA234.1, and the mutant strains *ΔagsA* and *ΔcrhA-G* showed a growth phenotype of compact pellets of slightly different sizes. In contrast, the deletion of *agsE* resulted in dispersed homogenous growth characteristics with a visible decrease in viscosity over time. Dispersed mycelial growth was characterized by reduced aggregation of germlings and was consistent across different cultivation media and multiple replicates. For better quantification, the particle size of the mycelial micro-colonies in CM liquid cultivation was analyzed in more detail (Fig. 9). The deletion of *agsA* resulted in the most similar particle size compared to the parental strain, whereas the smallest and most irregular pellets were quantified for all the analyzed *A. niger* strains lacking *agsE*. The knockout of the *crh* genes also resulted in a reduced pellet size phenotype, but not as strongly as that of the *agsE*-lacking strains.

In summary, we demonstrated that the deletion of *agsE* had the greatest impact on growth morphology in liquid culture, whereas the *agsA* and *crh* gene families did not alter growth morphology under growth conditions without applying any stressors. Moreover, the deletion of the entire *ags* gene family led to visible changes in colony morphology on agar plates and resulted in a drastically reduced and compact colony characteristic when combined with the *Δcrh* gene family.

## 4 Discussion

This study aimed to investigate the role of α-1,3-glucan synthases in *A. niger* for maintaining cell wall integrity and the fungus' ability to survive multiple deletions in cell wall biosynthetic gene families. *A. niger* possesses five *ags* gene copies, likely because of functional diversification or redundancy. As previously reported, *agsE* emerged as the most actively expressed gene, while *agsA* was identified as being highly upregulated in response to cell wall stress (Damveld et al., 2005). The differential expression profiles of these genes suggest distinct roles among the *ags* genes, and each gene appears to contribute uniquely to cell wall biosynthesis, structure, and dynamics under varying growth conditions. In our study of expression data and GO enrichment analysis, we identified several GO terms and genes with significant over-representation for the different *ags* genes, supporting the idea that these *ags* genes are expressed under different conditions. The majority of these are linked to the synthesis and modification of biological molecules essential for cellular processes, growth, and maintenance. However, the function of *agsB* and *agsC* remains unclear as we could not identify over-represented GO terms that were directly related to α-1,3-glucan biosynthesis or any other biological process.

By exploring *ags* single, double, and multiple knockout mutants of the entire gene family, we uncovered complex regulatory mechanisms and compensatory responses within the cell wall biosynthesis machinery. For instance, sensitivity assays have demonstrated that *ΔagsA* is less sensitive to cell wall stress than *ΔagsE* and *ΔagsA-E*.

Growth morphology studies have shown that *agsE* and even more *crhA-G* deletions seem to reduce the radial extension rate of *A. niger* strains on solid media, which would be a disadvantage for industrial protein or organic acid production strains. However, the deletion of *agsE* led to a unique, dispersed, and homogenously growing phenotype in liquid cultures with reduced germling aggregation and significantly smaller, irregular particle sizes, which could be highly advantageous for industrial applications.

Deletion of the three α-1,3-glucan synthases in *A. oryzae* led to smaller hyphal pellet formation, which allowed higher biomass formation and increased protein production (Miyazawa et al., 2016; Miyazawa et al., 2019; Yoshimi et al., 2017). Smaller micro-colonies of the *ΔagsE A. niger* have been shown to release more proteins than large micro-colonies but can be more sensitive to shear stress in liquid cultivation (Lyu et al., 2023a, Lyu et al., 2023b).

However, our *ags-*knockout strains, particularly *ΔagsE*, did not exhibit increased secretion of homologous glucoamylase (Fig. S7). Disperse growth characteristics have already been described for α-1,3-glucan synthase deletion mutants of other *Aspergillus* species (Yoshimi et al., 2013, Yoshimi et al., 2017; He et al., 2014; Miyazawa et al., 2016; Miyazawa et al., 2019; Miyazawa et al., 2020; Miyazawa et al., 2024); and our results showed that it is specifically linked to *agsE* in *A. niger*.

Remarkably, our study shows as consistent fact throughout that Ags proteins are not essential for survival under non-stress growth conditions. Our findings are consistent with those of previous studies suggesting that other components of the cell wall biosynthesis machinery can mitigate reduced α-1,3-glucan synthase activity (Yoshimi et al., 2013, Yoshimi et al., 2017; Lyu et al., 2023b).

Our investigation of *crh* gene family deletions also aligns with the results of previous studies. Significant alterations in the cell wall integrity of *A. niger* were only evident when the *crh* gene family deletion was combined with the reduction of α-glucan or galactomannan by deleting the respective *agsE* or *ugmA* (van Leeuwe et al., 2020). Interestingly, the *A. niger* strain lacking *the ags* and *crh* gene families showed a synergistic effect in our study compared to the *Δags* gene family alone.

Notably, antifungal drugs targeting specific enzymatic activities in cell wall biosynthesis did not affect *ags*, *crh*, or the double (*ags/crh*) mutants. Furthermore, protoplastation remained effective, and monosaccharide content and cell wall surface properties were not significantly altered, despite multiple gene deletions. Clearly, these results suggest unknown robust compensatory mechanisms but also indicate that α-glucan is not required to compensate for the loss of chitin-β-glucan cross-links in the *ΔcrhA-G* mutant.

However, the most significant changes in colony morphology were observed in the 12-fold knockout mutant, including reduced radial growth and sporulation defects indicating that the simultaneous lack of α-glucan and chitin-glucan crosslinking has consequences for the fitness of *A. niger*.

The fungal cell wall contains a β-1,3-glucan-chitin core, which is well established as a crucial structural component in most fungal species (Latgé, 2010; Free, 2013; Gow and Munro, 2017). In the cell wall of *A. fumigatus,* high-resolution techniques such as solid-state NMR spectroscopy have revealed that α-1,3-glucan contributes to the rigid hydrophobic cell wall core as a physical binding partner or another cross-link partner to β-1,3/β-1,6-glucans and chitin (Kang et al., 2018; Zhao et al., 2020; Chakraborty et al., 2021), but there is no evidence for covalently linked α-1,3-glucan-chitin or α-1,3-glucan-β-glucan in the cell walls of *A. niger*. Ags-encoding enzymes are associated with post-hyphal multicellularity, indicating their involvement in later stages of fungal growth (Kiss et al., 2019).

In contrast, Crh enzymes have been linked to hyphal evolution, suggesting their role in the initial phases of fungal development (Kiss et al., 2019). The diverse roles of *ags* and *crh* genes during different developmental stages underline their importance in maintaining cell wall integrity in fungi.

Our findings contribute to a deeper understanding of fungal cell wall dynamics and offer potential strategies for developing novel antifungal therapies, improving industrial fungal fermentation processes, managing fungal resistance in agriculture, and enhancing biotechnological applications involving fungal enzymes.

## 5 Conclusions

The fungal cell wall is vital for fungal survival and adaptability, and offers both strength and protection. Despite its crucial role, much of its dynamic composition and complex functions remain unclear. By investigating the effects of single and multiple deletions of α-1,3-glucan synthases in *A. niger,* alone and in combination with the *crh* gene family, we aimed to elucidate their roles in biosynthetic processes or pathways and their impact on fungal development and morphology.

A predicted diversity in functional roles among α-1,3-glucan synthases underscores the co-expression patterns of the *ags* genes within the complex network of cell wall biosynthesis and remodeling in *A. niger.* Our study highlights the impact of the *agsE* gene on maintaining the integrity of fungal cell walls through a unique growth phenotype and its related adaptive capabilities. Interestingly, the effects of *agsE* gene deletion do not directly alter the cell wall structure but lead to the recognition of a more complex interplay between cellular structure and function. The extensive deletion of multiple cell wall biosynthetic genes in *A. niger* indicates that the generated mutants must obtain survival strategies, such as compensatory responses, at the molecular level.

While the study provides substantial insights into fungal biology and offers potential targets for biotechnological applications, such as industrial fermentation processes for protein production, it also identifies additional research that requires further investigation.

Relationships between gene deletions, growth characteristics, and cell wall architecture enhance our understanding of the roles of specific genes and their encoding enzymes in fungal cell wall dynamics. Ongoing research is crucial to understand how fungal cells adapt, reorganize their cell walls, and maintain their integrity in response to environmental interactions.

The following are the supplementary data related to this article.

Supplementary material 1 Image 1

Supplementary material 2 Image 2

Supplementary material 3 Image 3

## Declaration of generative AI and AI-assisted technologies in the writing process

During the preparation of this work, the authors used AI assistance to enhance writing quality, including ChatGPT (GPT-4), Paperpal for text editing, and Grammarly for language corrections. After using these tools/services, the authors reviewed and edited the content as needed and take full responsibility for the content of the publication.

## Funding

This research did not receive any specific grant from funding agencies in the public, commercial, or not-for-profit sectors. Katharina Ost's position at the Osnabrück University of Applied Sciences was financed by funds from the Lower Saxony Ministry for Science and Culture (MWK) as part of the “Professorinnen für Niedersachsen” research program granted to Mareike E. Dirks-Hofmeister.

## Data linking

https://fungidb.org/fungidb/app/record/dataset/DS_bb15199826

## CRediT authorship contribution statement

**Katharina J. Ost:** Writing – original draft, Visualization, Validation, Methodology, Investigation, Formal analysis, Conceptualization. **Mark Arentshorst:** Investigation, Supervision. **Bruno M. Moerschbacher:** Resources, Supervision, Writing – review & editing. **Mareike E. Dirks-Hofmeister:** Methodology, Resources, Supervision, Writing – review & editing. **Arthur F.J. Ram:** Writing – review & editing, Supervision, Resources, Project administration, Funding acquisition, Data curation, Conceptualization.

## Declaration of competing interest

The authors declare that they have no known competing financial interests or personal relationships that could have appeared to influence the work reported in this paper.
